# Decoding the massive genome of loblolly pine using haploid DNA and novel assembly strategies

**DOI:** 10.1186/gb-2014-15-3-r59

**Published:** 2014-03-04

**Authors:** David B Neale, Jill L Wegrzyn, Kristian A Stevens, Aleksey V Zimin, Daniela Puiu, Marc W Crepeau, Charis Cardeno, Maxim Koriabine, Ann E Holtz-Morris, John D Liechty, Pedro J Martínez-García, Hans A Vasquez-Gross, Brian Y Lin, Jacob J Zieve, William M Dougherty, Sara Fuentes-Soriano, Le-Shin Wu, Don Gilbert, Guillaume Marçais, Michael Roberts, Carson Holt, Mark Yandell, John M Davis, Katherine E Smith, Jeffrey FD Dean, W Walter Lorenz, Ross W Whetten, Ronald Sederoff, Nicholas Wheeler, Patrick E McGuire, Doreen Main, Carol A Loopstra, Keithanne Mockaitis, Pieter J deJong, James A Yorke, Steven L Salzberg, Charles H Langley

**Affiliations:** 1 Department of Plant Sciences, University of California, Davis CA, USA; 2 Department of Evolution and Ecology, University of California, Davis CA, USA; 3 Institute for Physical Sciences and Technology (IPST), University of Maryland, College Park MD, USA; 4 Center for Computational Biology, McKusick-Nathans Institute of Genetic Medicine, Johns Hopkins University, Baltimore MD, USA; 5 Children’s Hospital Oakland Research Institute, Oakland CA, USA; 6 Department of Biology, Indiana University, Bloomington IN, USA; 7 National Center for Genome Analysis Support, Indiana University, Bloomington IN, USA; 8 Eccles Institute of Human Genetics, University of Utah, Salt Lake City UT, USA; 9 School of Forest Resources and Conservation, Genetics Institute, University of Florida, Gainesville FL, USA; 10 Southern Institute of Forest Genetics, USDA Forest Service, Southern Research Station, Saucier MS, USA; 11 Warnell School of Forestry and Natural Resources, University of Georgia, Athens GA, USA; 12 Department of Forestry and Environmental Resources, North Carolina State University, Raleigh NC, USA; 13 Department of Horticulture, Washington State University, Pullman WA, USA; 14 Department of Ecosystem Science and Management, Texas A&M University, College Station TX, USA

## Abstract

**Background:**

The size and complexity of conifer genomes has, until now, prevented full genome sequencing and assembly. The large research community and economic importance of loblolly pine, *Pinus taeda* L., made it an early candidate for reference sequence determination.

**Results:**

We develop a novel strategy to sequence the genome of loblolly pine that combines unique aspects of pine reproductive biology and genome assembly methodology. We use a whole genome shotgun approach relying primarily on next generation sequence generated from a single haploid seed megagametophyte from a loblolly pine tree, 20-1010, that has been used in industrial forest tree breeding. The resulting sequence and assembly was used to generate a draft genome spanning 23.2 Gbp and containing 20.1 Gbp with an N50 scaffold size of 66.9 kbp, making it a significant improvement over available conifer genomes. The long scaffold lengths allow the annotation of 50,172 gene models with intron lengths averaging over 2.7 kbp and sometimes exceeding 100 kbp in length. Analysis of orthologous gene sets identifies gene families that may be unique to conifers. We further characterize and expand the existing repeat library based on the *de novo* analysis of the repetitive content, estimated to encompass 82% of the genome.

**Conclusions:**

In addition to its value as a resource for researchers and breeders, the loblolly pine genome sequence and assembly reported here demonstrates a novel approach to sequencing the large and complex genomes of this important group of plants that can now be widely applied.

## Background

Advances in sequencing and assembly technologies have made it possible to obtain reference genome sequences for organisms once thought intractable, including the leviathan genomes (20 to 40 Gb) of conifers. Gymnosperms, represented principally by a diverse and majestic array of conifer species (approximately 630 species, distributed across eight families and 70 genera [1]), are one of the oldest of the major plant clades, having arisen from ancestral seed plants some 300 million years ago. Conifers will likely provide many genome-level insights on the origins of genetic diversity in higher plants.

Though today’s conifers may be considered relics of a once much-larger set of taxa that thrived throughout the age of the dinosaurs (250 to 65 millions of years ago) [2,3], they remain the dominant life forms in many of the temperate and boreal ecosystems in the Northern Hemisphere and extend into subtropical regions and the Southern Hemisphere.

We chose to investigate the loblolly pine (*Pinus taeda* L.) genome because of its well-developed scientific resources. Over 1.5 billion seedlings are planted annually, approximately 80% of which are genetically improved, driving its selection as the reference conifer genome. Among conifers, its genetic resources are unsurpassed in that three tree improvement cooperatives have been breeding loblolly pine for more than 60 years and manage millions of trees in genetic trials. The current consensus reference genetic map for loblolly pine is made up of 2,308 genetic markers [4]. Extensive QTL and association mapping studies in loblolly pine have revealed a great deal about the genetic basis of complex traits such as physical and chemical wood properties, disease and insect resistance, growth, and adaptation to changing environments. Current research focuses on the potential of genomic selection for continued genetic improvement [5].

The tree selected for sequencing, ‘20-1010’, is a member of the North Carolina State University-Industry Cooperative Tree Improvement Program and the property of the Commonwealth of Virginia Department of Forestry, which released this germplasm into the public domain. In accordance with open access policies [6], we released the first draft genome of loblolly pine in June 2012, which made it the first draft assembly available for any gymnosperm. The draft described here represents a significant advance over available gymnosperm reference sequences [7,8].

## Results and discussion

### Sequencing and assembly

The loblolly pine genome [9] joins the two other conifer reference sequences produced recently [7,8]. With an estimated 22 billion base pairs [10], it is the largest genome sequenced and assembled to date. Our experimental design leveraged a unique feature of the conifer life cycle and new computational approaches to reduce the assembly problem to a tractable scale [9,11]. From the first whole genome shotgun (WGS) assembly of the 1.8 million base pair *Haemophilus influenzae* genome in 1995 to the orders-of-magnitude larger three-billion-base-pair mammalian genomes that followed years later [12], the WGS protocol has been an efficient and effective method of producing high quality reference genomes. This was in part made possible by the overlap layout consensus (OLC) assembly paradigm championed by Myers [13] and ubiquitously implemented in first-generation WGS assemblers. When next-generation sequencing disruptively ushered in a new era of WGS sequencing, the extremely large numbers of reads exceeded the capabilities of existing OLC assemblers. To circumvent this, new assemblers were developed, using short k-mer based methods first described by Pevzner [14]. The giant panda [15] was the first mammalian species to have its genome produced using strictly NGS reads. For loblolly pine, we utilized a hybrid assembly method that incorporates both k-mer based and OLC assembly methods.

**Figure 1:**
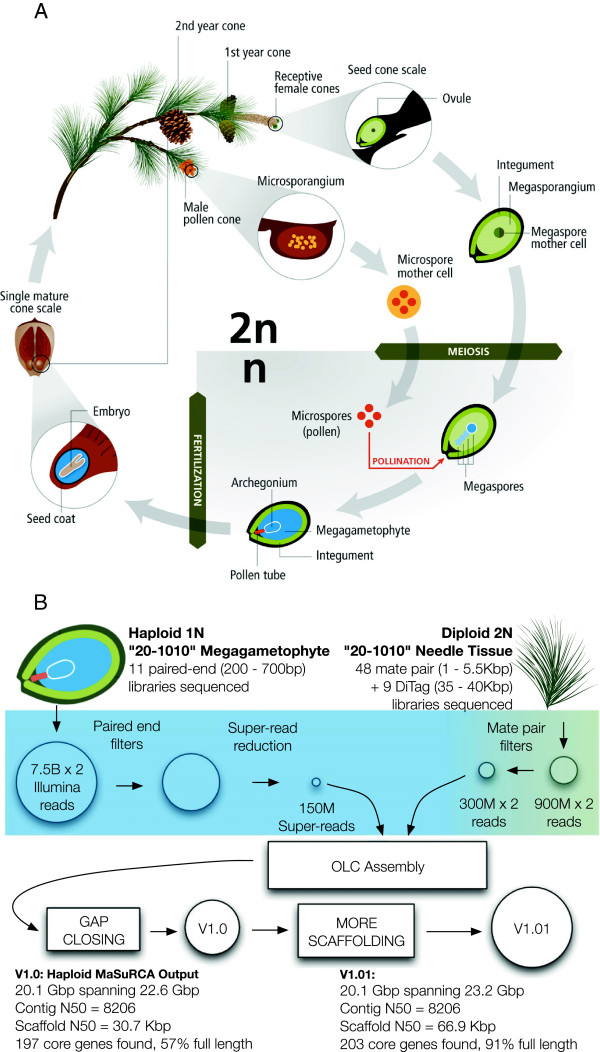
**(A) The sources of haploid and diploid genomic DNA.** The reproductive cycle of a conifer showing the unique sources of haploid and diploid genomic DNA sequenced. Both the ova pronucleus and the megagametophyte are derived by mitotic divisions from a single one of the four haploid meiotic segregant megaspores. The tissue from a single megagametophyte formed the basis for all of our shorter insert paired end Illumina libraries (Table 1). To construct longer insert libraries (Illumina mate pair and Fosmid DiTag) requiring greater amounts of starting DNA, needles from the parental genotype (20-1010) were used. **(B)** Sequencing and assembly schematic. An overlap layout consensus assembly, made possible by MaSuRCA’s critical reduction phase, was followed by additional scaffolding, incorporating transcript assemblies, to improve contiguity and completeness [9,11].

Figure 1A illustrates the two sources of DNA that comprised the sequencing strategy. As outlined below (see [9] for details), the majority of the WGS sequence data in Table 1 was generated from a single pine seed megagametophyte. The small quantity of genomic DNA obtained from the haploid megagametophyte tissue was used to construct a series of 11 Illumina paired end libraries with sufficient complexity to form the basis of a high quality WGS assembly. The use of haploid DNA greatly simplifies assembly, but the limited quantity of haploid DNA was insufficient for the entire project. Diploid needle tissue served as an abundant source of parental DNA for the construction of long-insert linking libraries. This included 48 libraries ranging from 1 to 5.5 kilobase pairs (Kb) and nine fosmid DiTag libraries spanning 35 to 40 Kb.

**Table 1:** Characteristics of the loblolly pine v1.01 draft assembly

**Estimated 1 N genome size**	**22 Gbp **[10]
Number of chromosomes	12
G + C%	38.2%
Sequence in contigs >64 bp	20,148,103,497 bp
Total span of scaffolds	23,180,477,227 bp
Contig N50	8,206 bp
Scaffold N50	66,920 bp
Haploid paired end libraries 200-600 bp	11 libraries
	7.5x billion x 2 reads (GA2x + HiSeq + MiSeq)
	1.4 trillion bp total read length
	63x sequence coverage
	150 million maximal super-reads
	52 billion total bp
	2.4x sequence coverage
Diploid mate pair libraries 1,000-5,500 bp	48 libraries
	863 million x 2 reads (GA2x)
	273 billion total read length
	270 million x 2 reads after filtering
	37x physical coverage
DiTag libraries 35-40 Kbp	9 libraries
	46 million x 2 reads (GA2x)
	4.5 million reads x 2 after filtering
	7.5x physical coverage

An overview of the assembly process is presented in Figure 1B. The combined 63× coverage from megagametophyte libraries (approximately 15 billion reads) was used for error correction and for the construction of a database of 79-mers appearing in the haploid genome. This database was used to filter highly divergent haplotypes from the diploid sequence data. The super-read reduction implemented in the MaSuRCA assembler [11] condensed most of the haploid paired-end reads into a set of approximately 150 M longer ‘super-reads’. Each super-read is a single contiguous haploid sequence that contains both ends of one or more paired-end reads. The construction process ensured that no super-read was contained in another super-read. Critically, the number of megagametophyte-derived reads was reduced by a factor of 100. The combined dataset was 27-fold smaller than the original, and was sufficiently reduced in size to make overlap-based assembly using CABOG [16] possible. The output of the MaSuRCA assembly pipeline became assembly 1.0. Additional scaffolding methods were implemented to improve the assembly by taking advantage of the deeply sampled transcriptome data [[17]], ultimately producing assembly v1.01. Finally, to further assess completeness, a scan for the 248 conserved core genes in the CEGMA database [18] was performed on all conifer assemblies (Figure 1B). The resulting annotations are classified as full length and partial. The loblolly pine v1.01 assembly has the largest number of total annotations (203) of the three conifers as well as the largest fraction of full length annotations (91%).

For validation purposes, we used a large pool of approximately 4,600 fosmid clones to approximate a random sample of the genome [9]. The sequenced and assembled pool contained 3,798 contigs longer than 20,000 bp, each putatively representing more than half of a fosmid insert, with a total span of 109 Mbp. When aligned to the genome 98.63% of the total length of these contigs was covered by the WGS assembly. A total of 2,120 of the aligned contigs had 99.5% or higher similarity, implying a combined error rate of less than 0.5%.

### Annotation

**Figure 2:**
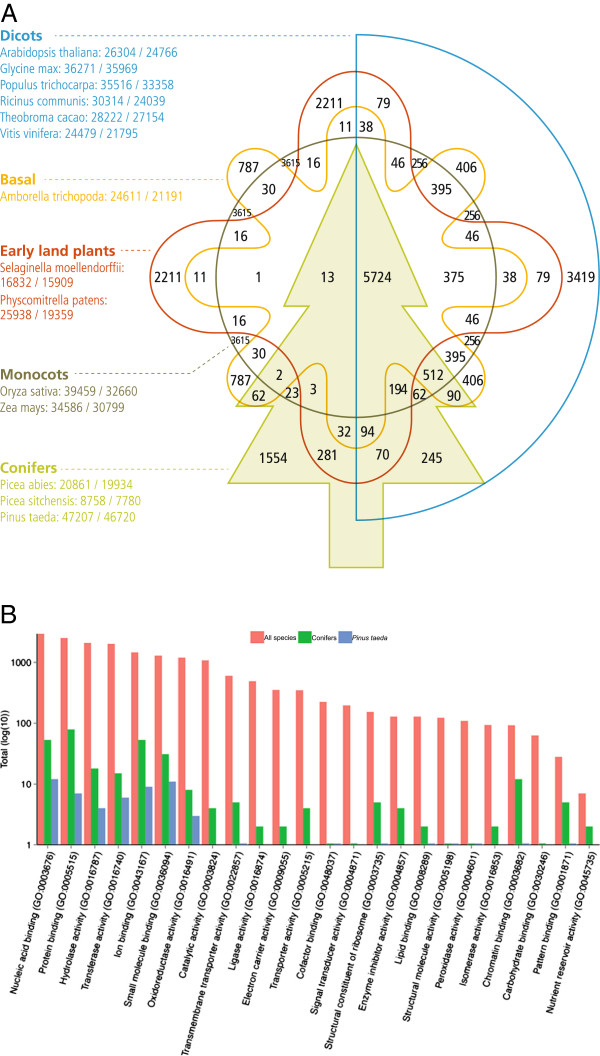
**Unique gene families and Gene Ontology term assignments. (A)** Identification of orthologous groups of genes for 14 species split into five categories: conifers (*Picea abies*, *Picea sitchensis*, and *Pinus taeda*), monocots (*Oryza sativa* and *Zea mays*), dicots (*Arabidopsis thaliana*, *Glycine max, Populus trichocarpa*, *Ricinus communis*, *Theobroma cacao*, and *Vitis vinifera*), early land plants (*Selaginella moellendorffii* and *Physcomitrella patens*), and a basal angiosperm (*Amborella trichopoda*). Here, we depict the number of clusters in common between the biological categories in the intersections. The total number of sequences for each species is provided under the name (total number of sequences/total number of clustered sequences). **(B)** Gene ontology molecular function term assignments by family for all species (red), conifers (green), and *Pinus taeda* exclusively (blue).

A *de novo* transcriptome assembly of 83,285 unique, full-length contigs from several tissue types and existing nucleotide resources (ESTs and conifer transcriptomes) supported a set of 50,172 unique gene models, derived from the MAKER-P annotation pipeline (Table 2) [19,20]. From the *de novo* transcriptome assembly, 42,822 aligned uniquely (98% identity and 95% coverage) to the genome. Of the 45,085 re-clustered loblolly pine EST sequences, 27,412 aligned (98% identity and 98% coverage). The frequent occurrence of pseudogenes (gene-like fragments representing 2.9% of the genome), required the use of conservative filters to define the final gene space [20]. The selected models represent coding-sequence lengths between 120 bp and 12 Kbp. Gene and exon lengths were comparable with angiosperm species; however, the number of full-length genes identified, even in a more fragmented genome, was greater than in other species (Figure 2A). Introns numbered 144,579 with an average length of 2.7 Kbp and a maximum length of 318 Kbp. A total of 6,267 (4.4%) of the introns were greater than 20 Kbp in length. This distribution far exceeds the intron lengths reported in other plant species and is, on average, longer than estimates in *Picea abies*[8,20]. The final gene models were identified on 31,284 scaffolds that were at least 10 Kbp in length. A total of 3,835 scaffolds contained three or more genes. Given the fragmentation of the genome and long intron lengths observed, it is likely that the genome contains additional genes, but also that some of the 50,172 models defined here may later be merged together.

**Table 2:** Comparison of gene metrics among sequenced plant genomes

	***Pinus taeda***	***Picea abies *** [8]	***Arabidopsis thaliana *** [21]	***Populus trichocarpa *** [21]	***Vitis vinifera *** [21]	***Amborella trichopoda *** [22]
**Genome size (assembled) (Mbp)**	20,148	12,019^a^	135	423	487	706
**Chromosomes**	12	12	5	19	19	13
**G + C content (%)**	38.2	37.9	35.0	33.3	36.2	35.5
**TE content (%)**	79	70	15.3	42	41.4	N/A
**Number of genes**^**b**^	50,172	58,587^c^	27,160	36,393	25,663	25,347
**Average CDS length (bps)**	965	723	1102	1143	1095	969
**Average intron length (bps)**	2,741	1,020	182	366	933	1,538
**Maximum intron length (bps)**	318,524	68,269	10,234	4,698	38,166	175,748

^a^Estimated genome size is 19.6 Gbp.

^b^Number of full-length genes >150 bp in length and validated through current annotations.

^c^High and medium confidence genes from the Congenie project [8].

We clustered the protein sequences in order to identify orthologous groups of genes [23]. Comparisons with 14 species, ignoring transposable elements, yielded 20,646 gene families with two or more members and 1,476 gene families present in all species (Figure 2A). Of the full set, 1,554 were specific to conifers and 159 of those were specific to loblolly pine [20].

The majority of characterized plant resistance proteins (R proteins) are members of the NB-ARC and NB-LRR families, and are associated with disease resistance [24]. Several independent families were identified containing one or both of these domains. The largest contained 43 loblolly pine members and 14 spruce members. Several other smaller families contained members exclusively from loblolly pine ranging from two to five members each. Other gene families with roles in disease resistance were also identified, including Chalone synthases (CHS) (three in loblolly pine and one spruce member). Increased expression of CHS is associated with the salicylic acid defense pathway [25].

Response to environmental stress, such as salinity and drought, has been investigated at length in conifers. Three different sets of Dehydrin (DHN) domains were noted, the largest with 10 loblolly pine members and 12 spruce members. The first genetic evidence of dehydrins playing a role in cellular protection during osmotic shock was in 2005, in *Physcomitrella patens*[26]. Subsequently, it was noted that transcription levels of a DHN increased in *Pinus pinaster* when exposed to drought conditions [27].

Cupins are members of a large, diverse family belonging to the Germin and Germin-like superfamily (GLP) [28]. In this analysis, several families, including one large family contained one or more domains related to Cupin 1. The largest family contained 23 loblolly pine members and five spruce members. These genes, similar to other GLPs, are expressed during somatic embryogenesis in conifers [28]. Cupins have therefore been associated with plant growth, and more recently associated with disease resistance in rice [29].

The *COPI C* family (58 members) was the largest exclusively identified in loblolly pine. Vesicle coat protein complexes containing *COPI* family members mediate transport between the ER and golgi, and interact with Ras-related transmembrane proteins, p23 and p24 [30]. Members of the Ras superfamily, *Arf* and *ArfGap*, also identified in loblolly pine, are involved in *COPI* vesicle formation [31]. These proteins were assigned to the small molecule binding GO category, which is enriched in pine and other conifers as compared with angiosperms. The other notable GO assignments include nucleic acid binding, protein binding, ion binding, and transferase activity which are consistent with the most populated categories for the other species included in the comparison (Figure 2B).

### Repetitive DNA content

**Figure 3:**
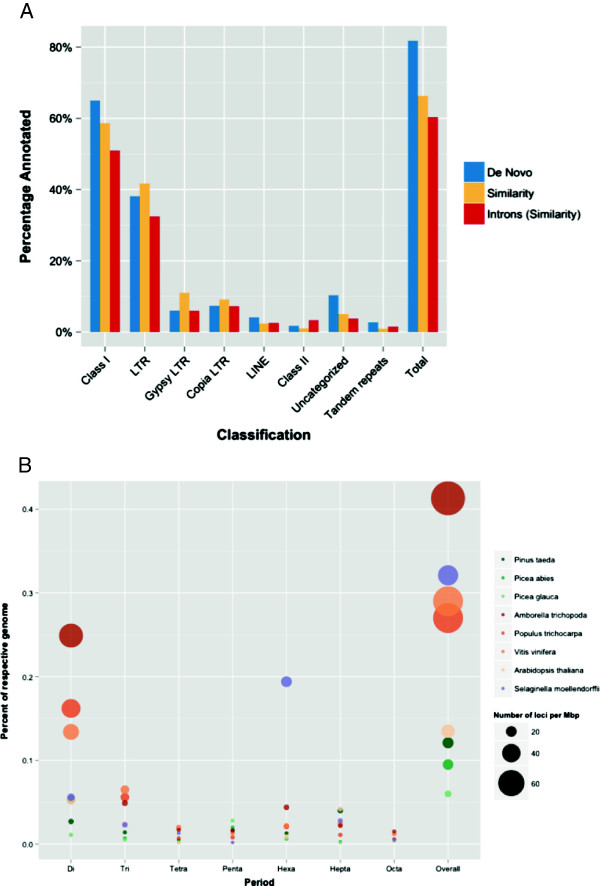
**Interspersed and tandem repetitive content. (A)** Overview of repetitive content in the *Pinus taeda* genome for similarity (blue) and *de novo* (yellow) approaches. Introns are evaluated with similarity methods against PIER 2.0 [32]. **(B)** Overview of microsatellite content across species with exclusion of mononucleotide repeats. Orange, green, and purple points represent angiosperm, gymnosperm, and lycophyte species, respectively. Each point displays both the density (point size) and length (y-axis) of di-, tri-, tetra-, penta-, hexa-, hepta-, and octanucleotide tandem repeats (x-axis). The *Overall* category is an accumulation of the previous seven categories.

Previous examination of the loblolly pine BAC and fosmid sequences led to the development of the Pine Interspersed Element Resource (PIER), a custom repeat library [32]. *De novo* analysis of 1% of the genome yielded 8,155 repeats, bringing PIER’s total to 19,194 [20]. The plethora of novel repeat content may be explained in part by the highly diverged nature of the repeat sequences, which prevents accurate identification from a reference library due to obscured similarities. Homology analysis demonstrated that retrotransposons dominated, representing 62% of the genome (Figure 3A). Seventy percent of these were long terminal repeat (LTR) retrotransposons. *PtConagree*[32] covered the largest portion of the genome, followed by *TPE1*[33], *PtRLC_3*, *PtRLX_3423*[20], *PtOuachita*, and *IFG7*[34]. Among introns, the estimated repetitive content was 60%. Introns were relatively rich in DNA transposons, at 3.31% (Figure 3A). Overall, the combined similarity and *de novo* approaches estimate that 82% of the pine genome is repetitive in nature (Figure 3A).

Though the genome is inundated with interspersed repetitive content, analysis revealed that only 2.86% is composed of tandem repeats, the majority of which are comprised of millions of retrotransposon LTRs. This estimate is comparable to the frequencies observed in other members of the Pineaceae (2.71% in *Picea glauca* (v1.0) and 2.40% in *Picea abies* (v1.0)) [20]. The number of tandem repeats may be dependent on sequencing and assembly methodologies. As shown previously, loblolly pine ranges from 2.57% in the WGS assembly [20] to 3.3% in Sanger-derived BACs [32]. Similar to most species, the relative frequencies of the repeating units of tandem loci are heavily weighted towards minisatellites (between 9 and 100 bp). This attribute is ubiquitous across plants but the smaller volume of microsatellites (1 to 9 bp repeating units) in conifers when compared to angiosperms and the increased contribution from heptanucleotides is significant (Figure 3B). A substantial number of loblolly pine’s tandem repeats are telomeric sequences (TTTAGGG)n (approximately 23,926 loci) and candidate centromeric sequences (TGGAAACCCCAAATTTTGGGCGCCGGG)n (5,183 loci, 1.8 Mbp). Originally identified in *Arabidopsis thaliana*[35], the telomeric sequences were found interstitially as well as at the end of the chromosomes in loblolly pine and other conifers [36]. The interstitial presence of the heptanucleotide repeat may explain the increased observation of this microsatellite in conifers. Pines have especially long telomeres, reaching up to 57 Kbp as found in *Pinus longaeva*[37,38].

### Organelle genomes

The mitochondrial genome was identified and assembled separately, taking advantage of its deeper coverage and distinctive GC content. The assembly was built primarily from 28.5 million high-quality 255-bp MiSeq reads generated during WGS sequencing. The read were first assembled with SOAPdenovo2 [39], and the resulting 7,559 scaffolds were aligned to the loblolly pine chloroplast genome [40] and to 557 complete and partial plant mitochondrial genomes. Twenty-seven scaffolds aligned to the chloroplast and 90 aligned to mitochondria. The mitochondrial scaffolds were distinguished by their coverage, which averaged >14x, while the coverage depth of chloroplast scaffolds was far deeper, more than 100x. The original reads represented just 0.3x coverage of the nuclear DNA. The mitochondrial GC-content was 44% *versus* 38.2% for the genome and 39.5% for the chloroplast. Based on these results, we identified 33 unaligned scaffolds longer than 1 Kb likely to be mitochondrial, with > = 44% GC content and coverage between 8x and 50x. These plus the 90 previously aligned scaffolds were reassembled, using additional reads from two WGS jumping libraries (lengths 3,800 bp and 5,200 bp), extracting only those pairs that matched the mitochondrial contigs. The resulting mitochondrial genome assembly has 35 scaffolds containing 40 contigs, with a total contig length of 1,253,551 bp and a maximum contig size of 256,879 bp.

### New insights in conifer functional biology

The draft genome sequence and transcriptome assemblies have enabled discovery of genes that underlie ecologically and evolutionarily important traits, illuminated larger-scale genomic organization of gene families, and revealed missing genes that evolved in angiosperms and not gymnosperms.

#### Disease resistance

The genome revealed that a partial EST containing a SNP was actually a candidate gene for rust resistance in loblolly pine. We mapped the SNP genetically, associated it with rust resistance then determined it was a toll-interleukin receptor/nucleotide binding/leucine-rich repeat (TNL) gene [41] containing signature domains only present in the new transcript and genome assemblies. Rust pathosystems can provide useful insights into host-pathogen co-evolution, because host resistance genes interact genetically with pathogen avirulence genes [42,43]. Analysis of fusiform rust pathogen *Cronartium quercuum* (Berk.) Miyabe ex Shirai f.sp. *fusiforme* (*Cqf*) genetic interactions with *Pinus* hosts [44,45] led to mapping of *F*usiform *r*ust resistance *1* (*Fr1*; [46]) to LG2 [47,48]. *P. lambertiana Cr1* for white pine blister rust resistance [49] was also mapped to the same linkage group using syntenic markers [50].

**Figure 4:**
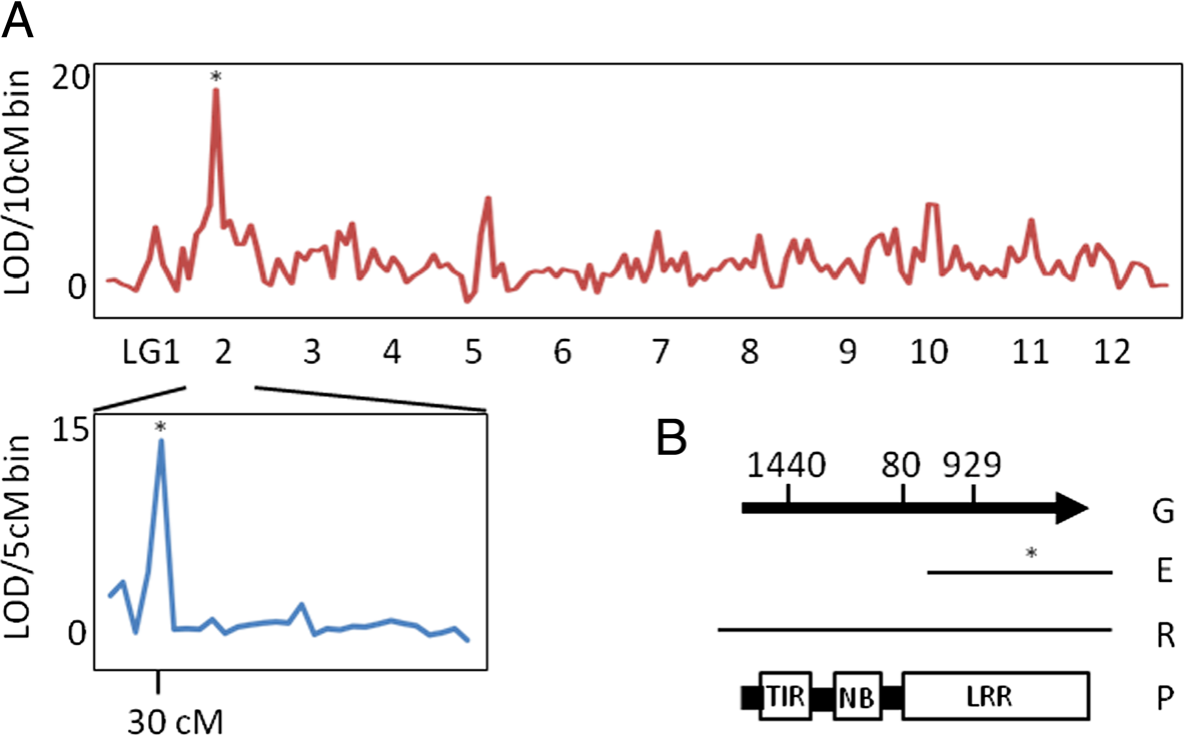
**Identification of TNL candidate gene for Fr1. (A)** Genome survey of rust resistance in segregating progeny of Fr1/fr1 *Pinus taeda* among clonally propagated half-siblings (upper) and full-siblings (lower). Bins with highest LOD scores contained. SNP 2_5345_01 (*). **(B)** Translated gene model (G) on genome scaffold jcf7180063178873 is interrupted by three introns with sizes given in bp, previously available EST (E) containing SNP 2_5345_01 (*), fully assembled transcript Evg1_1A_all_VO_L_3760_240252 from RNAseq (R) and the domain structure of the protein model (P).

Two large mapping populations were used to assign genetic map positions to 2,308 SNP markers that were mapped to genomic scaffolds [4], whose SNPs were then tested for association with rust resistance [48] in a family-based, clonal population [51]. The top-ranked SNP for rust resistance in a parent segregating for *Fr1* mapped to LG2 (31.3 cM; Figure 4A) and occurred in a transcript model encoded by a TNL-type gene located on a genomic scaffold (Figure 4B). The TNL-type gene is related to *N* from *Nicotiana*[52] that belongs to a large class of genes for resistance to biotrophic pathogen-induced diseases [41,53]. Prior to this work the loblolly pine gene product appeared to lack TIR and NB domains because the EST was truncated. Based on OrthoMCL analysis of the full-length proteins [20], the gene belongs to a class of TNLs that have expanded in conifers (N = 780 in loblolly pine; N = 180 in *P. abies*) but not *Arabidopsis* (N = 3). By contrast, most TNL genes in *Arabidopsis* belong to a large class (N = 138) not found in loblolly pine or *P. abies*.

The genome sequence has revealed that distinct classes of TNLs have expanded in conifers and angiosperms, making it feasible to test conifer candidate genes for co-segregation with disease resistance, instead of using markers derived from incomplete ESTs or other species. The transcript of the TNL gene is detected in young stems, reaction wood, in hymenial layers obtained from fusiform rust galls, and is a candidate for *Fr1. Avr1*, the avirulence gene that specifically interacts with *Fr1*[54], has been genetically mapped on LGIII of *Cqf* and the genome sequence is now available [55]. These genome-based discoveries open the door to understanding the effects of host *Fr* genes on allelic diversity and frequency in their corresponding *Avr* genes, and vice versa, at large geographic scales. The practical outcomes for *Fr* gene durability are significant given the widespread planting of >500 million loblolly pine seedlings each year that harbor one or more fusiform rust resistance loci as a consequence of parental selection, selective breeding, and screening for fusiform rust resistance [56]. Comparisons of *Fr1* and *Cr1* loci should generate new insights into evolution of resistance genes [57] within a genus that arose 102-190 million years ago [58].

#### Stress response

Conifers dominate a variety of biomes by virtue of their capacity to survive and thrive in the face of extreme abiotic stresses. For example, pronounced resistance to water stress, particularly in mature trees, has enabled conifers to spread across deserts and alpine areas, well beyond the range of most competing woody angiosperms. At the same time, water stress is a major cause of mortality for conifer seedlings [59], and predictions hold that differential susceptibility of conifer species to water stress will have profound consequences for forest and ecosystem dynamics under future climate change scenarios [60]. Variation in drought resistance in conifers has long been recognized to have a genetic basis [61,62], and substantial effort has previously been devoted to attempts at using molecular and genomic tools to uncover the responsible genetic determinants [63-65].

One of the first drought-responsive conifer genes to be cloned and characterized was *lp3*[66], which was shown to share homology with a small family of nuclear-localized, ABA-inducible genes (termed *ASR* for ABA-, Stress- and Ripening) initially identified in tomato [67,68]. Subsequent work has shown that *ASR* genes are broadly distributed in higher plants and adaptive alleles of these genes are determinants of drought resistance in wild relatives of various domesticated crops [69-71]. Transcriptomic studies have detected differential expression of *lp3* gene family members in drought-stressed pine [72,73], while other studies have linked expression of *lp3* gene family members to aspects of wood formation, that is, xylem development [74,75] and cold tolerance [76]. Genetic studies indicate that *lp3* alleles are under selection in pine and likely confer adaptive resistance to drought [77,78].

**Figure 5:**
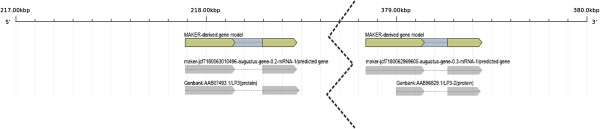
***Pinus taeda*** **lp3 sequences from Genbank (AAB07493, AAB96829) were aligned to the same scaffold in v1.01 and supported by two distinct MAKER-derived gene model.**

Protein sequences for the four distinct loblolly pine *lp3* gene family members in GenBank (AAB07493, AAB02692, AAB96829, AAB03388) aligned optimally to four of the high-confidence gene models. The *ASR* genes in tomato are physically clustered and have been held out as examples of tandemly arrayed genes that are important for adaptation [70]. In the v1.01 assembly, two of the pine *lp3* genes (AAB07493, AAB96829) were found to reside on the same scaffold (Figure 5). Very little is known about the physical clustering of gene family members in conifers, but the availability of the loblolly pine genome sequence now provides the opportunity to study such relationships and their contribution to adaptation in conifers.

#### Wood formation

The genome assembly and annotation provide new information on the roles of specific genes involved in wood formation. Pine secondary xylem contains large numbers of tracheids with abundant bordered pits for both mechanical support and water transport; by contrast, the secondary xylem of woody dicots typically has specialized vessel elements for water conduction, and fiber cells for mechanical support [79,80]. The chemical composition of gymnosperm xylem is characterized by a guaiacyl-rich (G type) lignin and the absence of syringyl (S type) subunits [81]. The lignins in the xylem of woody dicots, gnetales, and Selaginella (a lycopod) are characterized by a mixed polymer of S and G subunits (S/G lignin) [82]. The hemicelluloses of pines are a mixture of heteromannans, while dicot hemicelluloses are typically xylan-rich [83]. The functional and evolutionary differences in lignin composition, hemicellulose composition, and presence of vessel elements between gymnosperms and angiosperms are informed by the pine genome sequence. Expressed homologs of all but one of the known genes for lignin precursor (monolignol) biosynthesis [84] have been identified in the pine genome assembly. The exception is the gene encoding ferulate 5-hydroxylase (also called coniferaldehyde 5-hydroxylase), the key enzyme for the formation of sinapyl alcohol, the precursor for S subunits in S/G lignin [85]. The absence of a 5-hydroxylase homolog is significant because of the depth of pine sequencing and the quality of the annotation [20]. A putative homolog of a gene only recently implicated in monolignol biosynthesis, encoding caffeoyl shikimate esterase [86], has also been identified in the pine annotation. Some monolignol gene variants are associated with quantitative variation in growth and wood properties such as wood density or microfibril angle [87-89]. The draft pine genome assembly contains putative homologs of six cellulose synthase subunits (CesA1, 3, 4, 5, 7, and 8), and two putative gene models for glucomannan 4-beta-mannosyltransferases and two for xyloglucan glycosyltransferases, consistent with the hemicellulose composition of pine. The pine genome assembly also contains putative homologs for many genes that encode transcription factors that regulate wood cell types or the perennial growth habit [90,91]. This information is useful to guide the genetic improvement of wood properties as resources for biomaterials and bioenergy.

## Conclusions

The loblolly pine reference genome joins the recent genomes of Norway spruce and white spruce forming a foundation for conifer genomics. To tackle the problem of reconstructing reference sequences for these leviathan genomes, the three projects each used different approaches. The whole genome shotgun approach has been historically favored because it gives a rapid result. An alternative has been the expensive and time-consuming application of cloning to reduce to complexity of the problem for tractability or to obtain a better result [8]. Our combined strategy resulted in the most complete and contiguous conifer (gymnosperm) genome sequenced and assembled to date [9] with an assembled reference sequence consisting of 20.1 billion base pairs contained in scaffolds spanning 22.18 billion base pairs.

Our efforts to improve the quality of the loblolly pine reference genome sequence for conifers are continuing. The importance of a high quality and complete reference sequence for major taxonomic groups is well chronicled [92]. The loblolly pine reference genome was obtained from a single tree, 20-1010, for which significant and continuing open-access genome resources are freely available through the Dendrome Project and TreeGenes Database [93].

## Materials and methods

### Reference genotype tissue and DNA

All source material was obtained from grafted ramets of our reference *Pinus taeda* genotype 20-1010. Our haploid target megagametophyte was dissected from a wind-pollinated pine seed collected from a tree in a Virginia Department of Forestry seed orchard near Providence Forge, Virginia. Diploid tissue was obtained from needles collected from trees at the Erambert Genetic Resource Management Area near Brooklyn, Mississippi and the Harrison Experimental Forest near Saucier, Mississippi. A detailed description of the preparation and QC of DNA from these tissue samples is contained in [9].

### Sequencing, assembly, and validation

A detailed description of the whole genome shotgun sequencing, assembly, and validation of the V1.0 and V1.01 loblolly pine genomes is contained in [9].

To compare the contiguity of our V1.01 whole genome shotgun assembly to contemporary conifer genome assemblies the scaffold sequences for white spruce genome [7] and Norway spruce [8] were obtained from Genbank.

CEGMA analysis of the core gene set [18] performed on the V1.0 and V1.01 loblolly pine genomes was obtained as described in [9]. Similarly, a Norway spruce analysis was performed with results consistent with those reported in [8]. The results for the white spruce assembly were taken directly from [7].

To assemble the mitochondrial genome, a subset of the WGS sequence consisting of 255 bp paired end MiSeq reads from four Illumina paired end libraries (median insert sizes: 325, 441, 565, and 637) were selected for an independent organelle assembly. The 28.5 Mbp of sequence, representing less than 0.3× nuclear genomic coverage, was assembled using SOAPdenovo2 (K = 127). The resulting contigs were aligned using nucmer to a database containing the loblolly pine chloroplast, sequencing vector, 102 BACs, and 50 complete plant mitochondria. Contigs were identified and labeled as mitochondrial if they aligned exclusively to existing mitochondrial sequence and had high coverage (> = 8×) and G + C% (> = 44%). The contigs were then combined with additional linking libraries, the LPMP_23 mate pair library and all DiTag libraries, and assembled a second time with SOAPdenovo2. Subsequently intra-scaffold gaps were closed using and GapCloser (v1.12). The assembled sequences were iteratively scaffolded and gaps were closed until no assembly improvements could be made.

### Annotation

The assembled genome was annotated with the MAKER-P pipeline [19] as described in [20]. Prior to gene prediction, the sequence was masked with similarity searches against RepBase and the Pine Interspersed Element Resource (PIER) [32]. Following the annotation, the TRIBE-MCL pipeline [94], was used to cluster the 399,358 protein sequences from 14 species into orthologous groups as described in [20].

### Repetitive DNA content

Interspersed repeat detection was carried out in two stages, homology-based and *de novo* as described in [20]. For homology-based identification, RepeatMasker 3.3.0 [95] was run against the PIER 2.0 repeat library [32] for both the full genome and introns. REPET 2.0 [96] was implemented with the pipeline described in [32] for *de novo* repeat discovery. Only the 63 longest scaffolds were used in the all-vs-all alignment (approximately 1% of the genome). In addition, PIER 2.0, the spruce repeat database, and publicly available transcripts from *Pinus taeda* and *Pinus elliottii* were utilized as input for known repeat and host gene recognition.

To identify tandem repeats, Tandem Repeat Finder (v4.0.7b) [97] was run on both the genome and transcriptome as described in [20]. Filtering of multimeric repeats and overlaps with interspersed repeats, helped assess total tandem coverage and relative frequencies of specific satellites.

### Data availability

Primary sequence data may be obtained from NCBI and is indexed under BioProject PRJNA174450. The whole genome shotgun sequence obtained for this assembly is available from the sequence read archive (SRA: SRP034079). The V1.0 and V1.01 genome sequences are available at [98]. Access to gene models, annotations, and Genome Browsers [99,100] are available through the TreeGenes database [93,101].

## Competing interests

The authors declare that they have no competing interests.

## Authors’ contributions

DBN, JLW, KAS, AZ, DM, CAL, KM, PJDJ, JAY, SLS, and CHL designed the research. DBN, JLW, KAS, AZ, DP, MC, CC, MK, AEH, JDL, PJMG, HAVG, BYL, JJZ, WMD, SFS, LW, DG, GM, MR, CH, MY, JMD, KS, JFDD, WWL, RWW, RS, DM, CAL, KM, PJDJ, JAY, SLS, and CHL performed research and analyzed data. DBN, JLW, KAS, JMD, JFDD, RWW, RS, NW, PEM, CAL, SLS, and CHL wrote the article. All authors read and approved the final manuscript.
